# Near-Infrared Spectroscopy Combined with Fuzzy Improved Direct Linear Discriminant Analysis for Nondestructive Discrimination of Chrysanthemum Tea Varieties

**DOI:** 10.3390/foods13101439

**Published:** 2024-05-07

**Authors:** Jiawei Zhang, Xiaohong Wu, Chengyu He, Bin Wu, Shuyu Zhang, Jun Sun

**Affiliations:** 1 Mengxi Honors College, Jiangsu University, Zhenjiang 212013, China; 3210608050@stmail.ujs.edu.cn (J.Z.); 3210608017@stmail.ujs.edu.cn (S.Z.); 2 School of Electrical and Information Engineering, Jiangsu University, Zhenjiang 212013, China; 2222107003@stmail.ujs.edu.cn (C.H.); sun2000jun@ujs.edu.cn (J.S.); 3 High-Tech Key Laboratory of Agricultural Equipment and Intelligence of Jiangsu Province, Jiangsu University, Zhenjiang 212013, China; 4 Department of Information Engineering, Chuzhou Polytechnic, Chuzhou 239000, China

**Keywords:** chrysanthemum tea, near-infrared spectroscopy, dimensionality reduction, feature extraction

## Abstract

The quality of chrysanthemum tea has a great connection with its variety. Different types of chrysanthemum tea have very different efficacies and functions. Moreover, the discrimination of chrysanthemum tea varieties is a significant issue in the tea industry. Therefore, to correctly and non-destructively categorize chrysanthemum tea samples, this study attempted to design a novel feature extraction method based on the fuzzy set theory and improved direct linear discriminant analysis (IDLDA), called fuzzy IDLDA (FIDLDA), for extracting the discriminant features from the near-infrared (NIR) spectral data of chrysanthemum tea. To start with, a portable NIR spectrometer was used to collect NIR data for five varieties of chrysanthemum tea, totaling 400 samples. Secondly, the raw NIR spectra were processed by four different pretreatment methods to reduce noise and redundant data. Thirdly, NIR data dimensionality reduction was performed by principal component analysis (PCA). Fourthly, feature extraction from the NIR spectra was performed by linear discriminant analysis (LDA), IDLDA, and FIDLDA. Finally, the K-nearest neighbor (KNN) algorithm was applied to evaluate the classification accuracy of the discrimination system. The experimental results show that the discrimination accuracies of LDA, IDLDA, and FIDLDA could reach 87.2%, 94.4%, and 99.2%, respectively. Therefore, the combination of near-infrared spectroscopy and FIDLDA has great application potential and prospects in the field of nondestructive discrimination of chrysanthemum tea varieties.

## 1. Introduction

Chrysanthemum tea is a valuable flower crop in China, and it is widely used in traditional Chinese medicine for its high medicinal value [1]. It has many beneficial chemical components, including flavonoids, polysaccharides, and unsaturated fatty acids [2], as well as luteolin and luteoloside [3]. It has been proven that chrysanthemum tea can be used to fight cancer, inflammation, and obesity, protect the liver and kidneys, and guard against liver-fire hyperactivity syndrome [4]. The quality and efficacy of chrysanthemum tea are closely related to its geographical origin [5]. As a result, the market is susceptible to fraudulent substitutes of lower value, which would be detrimental to the health and interests of consumers. Therefore, it is crucial to develop a quick and effective method to identify the chrysanthemum tea varieties.

In recent years, many researchers have actively explored some identification methods for chrysanthemum tea varieties. For example, Luo et al. applied gas chromatography–mass spectrometry and olfactometry and an electronic nose combined with principal component analysis (PCA) to identify the geographical origins of Chinese chrysanthemum flower teas [6]. DNA barcoding analysis based on PsbA-trnH, matK, and trnl has been proven to be effective in the identification of chrysanthemum varieties living in different geographic populations [7]. Hao et al. successfully classified nine geographically distinct chrysanthemum varieties using laser-induced breakdown spectroscopy and chemometrics [8]. However, these techniques are complex in terms of data preprocessing and are relatively costly and slow, so they are unsuitable for the rapid non-invasive detection of chrysanthemum tea varieties.

Currently, near-infrared (NIR) spectroscopy technology is developing rapidly due to the advantages of miniaturized NIR spectrometers [9], and it has good application prospects in the field of nondestructive food detection with its advantages of simplicity, efficiency, and low cost [10,11,12,13,14]. Nowadays, the widespread application of NIR spectroscopy technology appears in the agriculture and food industry [15,16,17,18,19,20,21,22], chemical and material science [23], pharmaceutical industry, and many other fields [24]. For example, Ma et al. combined NIR spectroscopy with partial least squares and an artificial neural network for the rapid detection of sugarcane stalk bending characteristics [25]. Wu et al. utilized a novel fuzzy feature extraction algorithm to process the NIR data of Chunmee tea and established an effective classification model [26]. NIR spectroscopy was combined with chemometrics to identify different tea varieties, and the classification accuracy reached 98.33% in [27]. Chen et al. designed a classification method using NIR spectroscopy and a random forest algorithm to accurately classify tea quality [28].

NIR spectra are characterized by high dimensionality, overlap, and nonlinearity, so the accuracy is low if the NIR spectra are classified directly. A common solution is to first pretreat the NIR spectra and then perform feature extraction on the spectra. Feature extraction algorithms are important for solving small-sample-size (SSS) problems [29]. When linear discriminant analysis (LDA) processes NIR spectra with high dimensionality, SSS problems always arise. In recent years, many approaches have been proposed for solving this SSS problem [30,31,32], and one of them is direct LDA (DLDA). High-dimensional spectral data usually need to be downscaled by PCA, but some feature information may be lost in this process. The DLDA algorithm can avoid this problem as it can directly extract features from high-dimensional data [33]. But DLDA discards the zero space of the interclass scattering matrix in its computation, and the zero space may have useful information for categorization. This reduces the classification accuracy, and improved DLDA (IDLDA) was proposed to solve the drawbacks of DLDA [34]. However, the classification performance of the IDLDA algorithm may suffer from data overlap. To solve this problem, fuzzy IDLDA (FIDLDA) is proposed in this study to extract the NIR spectra of chrysanthemum tea.

Zadeh et al. introduced fuzzy set theory, which could be a good solution to the data overlap problem [35]. Some feature extraction algorithms have been combined with fuzzy set theory for spectral information extraction. Fuzzy improved null LDA (FiNLDA) was employed to attain the near-infrared spectral discrimination of milk, and an effective model for milk brand discrimination was developed [36]. Fuzzy uncorrelated discriminant transformation (FUDT) was utilized to process the NIR spectrum of milk and achieved a classification accuracy of 98.67% in identifying the geographical sources of milk [37]. Therefore, it is feasible to combine a fuzzy algorithm, feature extraction methods, and NIR spectroscopy for discriminative information extraction. In this experiment, a classification model using NIR spectroscopy and FIDLDA was designed for the nondestructive discrimination of chrysanthemum tea varieties.

## 2. Materials and Methods

### 2.1. Sample Preparation

Five types of chrysanthemum tea, including chuju (CJ), hangbaiju (HBJ), huaiju (HJ), huangshangongju (HSGJ), and wuyuanhuangju (WYHJ), originated from Chuzhou, Tongxiang, Jiaozuo, Huangshan, and Wuyuan in China, respectively. The distinguishing differences between these types were the contents of several functional components, which are shown in Table 1 [38,39]. The tea had a golden or light brown appearance, a clear odor, a good even size, no mold, and intact inflorescences. To keep them dry and cool, they were stored in sealed food preservation bags until NIR analysis was performed.

A total of 400 samples were used for the spectral data collection. The same number of samples were procured for each category, and they were divided into five groups according to their varieties, so each group had 80 samples. Subsequently, all chrysanthemum tea samples were partitioned into a training set and a test set based on a specific ratio in the discriminant experiment. Spectral acquisition was performed at about 20 °C and 60% relative humidity.

### 2.2. NIR Spectra Collection

The NIR spectra of the samples were collected in Hadamard mode using a portable spectrometer, NIR-M-F1-C (Shenzhen Puyan Internet Technology, Shenzhen, China). Using a spectrometer in Hadamard mode can improve the signal-to-noise ratio (SNR), and a higher optical energy can be captured by an InGaAs detector. The spectrometer operated at wavelengths from 900 to 1700 nm. The ratio of signal to noise and the optical resolution were set to 6000:1 and 10 nm for the acquisition process, respectively. The spectrometer was equipped with a humidity and temperature sensor. Each spectrum consisted of 400 data points with a wavelength interval of 800 nm.

**Figure 1:**
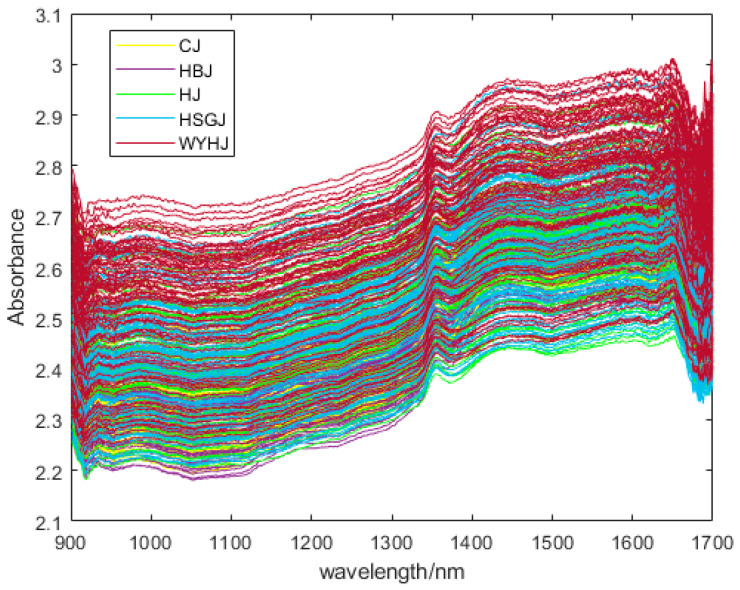
The raw spectra of chrysanthemum tea samples.

The scans were performed 8 times, and each scan had an exposure time of 0.625 ms. In this experiment, non-invasive reflectance detection was utilized. Figure 1 displays the raw NIR spectra of the chrysanthemum tea samples.

### 2.3. Preprocessing

By using a NIR-M-F1-C spectrometer to analyze the samples, the raw NIR spectra of the chrysanthemum tea varieties could be obtained. However, the direction of light changes due to the effect of small inhomogeneity on the surface of chrysanthemum tea when collecting spectral data, and the noise-generated scatter may affect the raw NIR spectra [40], and, therefore, preprocessing the spectral data is important for the subsequent processing of the NIR spectra. In this experiment, several preprocessing algorithms were applied to pretreat the NIR spectral data, including standard normal variation (SNV), multiplicative scattering correction (MSC), Savitsky–Golay (SG) filtering [41], and mean centering (MC), which improved the spectral data. Combined pretreatment methods were also tried, like SG + MSC and SG + SNV, but the effects were not very satisfactory in this experiment.

**Figure 2:**
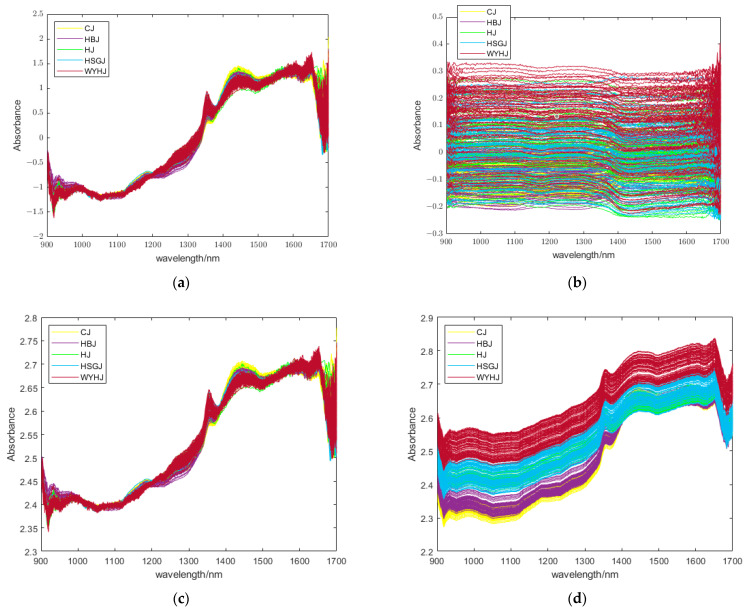
NIR spectra of chrysanthemum tea samples preprocessed by four pretreatment methods: (**a**) SNV, (**b**) MC, (**c**) MSC, and (**d**) S-G filtering. SNV, standard normal variation; MC, mean centering; MSC, multiplicative scattering correction; S-G, Savitsky–Golay.

MSC can reduce the negative effects of uneven particle sizes, optical path variations, varying sample compactness, and spectral noise and bias. SNV can correct scattering effects and baseline shifts in spectral data and reduce inter-sample variation. SG filtering can also remove the spectral noise and enhance the smoothness of spectral data. MC can improve the comparability between variables, amplify weak signals, and reduce the collinearity between spectral data. Figure 2 shows the NIR spectral data preprocessed by the four single pretreatment methods.

### 2.4. Data Processing Algorithms

#### 2.4.1. Principal Component Analysis

The collected infrared spectra of the chrysanthemum tea samples had a dimension of 400, which contained a large amount of redundant information and noisy data, which may increase the computational cost and decrease the classification accuracy. Therefore, to acquire high-quality spectral data, it was necessary to perform dimensionality reduction and redundancy removal on the pretreated spectral data. PCA is one of the commonly used methods, which operates by identifying a collection of orthogonal eigenvectors that make their corresponding eigenvalues as large as possible, and dimensionality can be reduced by choosing a meaningful set of eigenvectors. Because these eigenvectors correspond to larger variance, the most significant information in the raw data can be retained while reducing the dimensionality.

#### 2.4.2. LDA

LDA is a classical machine learning algorithm that is utilized for both the extraction of features and reducing data dimensionality [42]. LDA can reduce the complexity of spectral data by finding the most representative features in the data. In order to be able to distinguish between different classes of data, the primary objective of the LDA algorithm is to determine the ideal projection direction to make the inter-class spacing as large as possible and minimize the intra-class spacing as much as possible.

#### 2.4.3. IDLDA

IDLDA is another important technique for the extraction of features in the widespread use of small-sample problem solving [34]. The steps of IDLDA are described as follows (Algorithm 1).
**Algorithm 1:** The Steps of IDLDA AlgorithmStep 1. Build the matrices $S_{t}$, $S_{b}$, and $S_{w}$;  Step 2. Singular value decomposition of $S_{w}$ as $S_{w}=U_{w}{D_{w}}^{2}{U_{w}}^{T}$;  Step 3. Find $\partial =\max(diag(D_{w}))$ and build $D_{\partial }=\partial I_{d\times d}-D_{w}$;  Step 4. Use $D_{\partial }$ and $U_{w}$ to diagonalize $S_{fb}$, as $D_{\partial }{U_{w}}^{T}S_{b}U_{w}D_{\partial }=F\Sigma^{2}F^{T}$, where $F=\left[F_{r},F_{n}\right]$ such that $F_{r}$ corresponds to the range space of $S_{b}$ and $F_{n}$ corresponds to the null space of $S_{b}$;  Step 5. Calculate the transformation matrix $W_{IDLDA}=U_{w}D_{\partial }F_{r}$, and project samples into the feature space.

In Step 1, $S_{t}$ represents the total scatter matrix; $S_{b}$ represents the between-class matrix; and $S_{w}$ represents the within-class matrix. They are listed as follows:

(1) $$S_{t}=\sum_{i=1}^{n}\left(x_{i}-\bar{x}\right){\left(x_{i}-\bar{x}\right)}^{T}$$

(2) $$S_{b}=\sum_{j=1}^{c}n_{j}\left({\bar{x}}_{j}-\bar{x}\right){\left({\bar{x}}_{j}-\bar{x}\right)}^{T}$$

(3) $$S_{w}=\sum_{j=1}^{c}\sum_{x_{i}\in c_{i}}\left(x_{i}-{\bar{x}}_{j}\right){\left(x_{i}-{\bar{x}}_{j}\right)}^{T}$$

where n represents the sample number; $n_{j}$ is the sample number in the jth class; and c denotes the number of the variety.

#### 2.4.4. FIDLDA

FIDLDA is a novel fuzzy DLDA algorithm generated based on the combination of fuzzy set theory and the IDLDA algorithm. The specific algorithm execution steps are shown as follows (Algorithm 2).
**Algorithm 2:** The Steps of FIDLDA AlgorithmStep 1. Build the matrices $S_{ft}$, $S_{fb}$, and $S_{fw}$;  Step 2. Singular value decomposition of $S_{fw}$ as $S_{fw}=U_{fw}{D_{fw}}^{2}{U_{fw}}^{T}$;  Step 3. Find $\partial_{f}=\max(diag(D_{fw}))$ and build $D_{\partial_{f}}=\partial_{f}I_{d\times d}-D_{fw}$;  Step 4. Use $D_{\partial_{f}}$ and $U_{fw}$ to diagonalize $S_{fb}$, as $D_{\partial_{f}}{U_{fw}}^{T}S_{fb}U_{fw}D_{\partial_{f}}=F_{f}\Sigma^{2}{F_{f}}^{T}$, where $F_{f}=\left[F_{fr},F_{fn}\right]$ such that $F_{fr}$ corresponds to the range space of $S_{fb}$ and $F_{fn}$ corresponds to the null space of $S_{fb}$;  Step 5. Calculate the transformation matrix $W_{FIDLDA}=U_{fw}D_{\partial_{f}}F_{fr}$, and project samples into the feature space.

In Step 1, $S_{ft}$ represents the fuzzy total scatter matrix; $S_{fb}$ represents the fuzzy between-class matrix; and $S_{fw}$ represents the fuzzy within-class matrix. They can be calculated as follows:

(4) $$S_{ft}=\sum_{j=1}^{c}\sum_{i=1}^{n}u_{ij}^{m}\left(x_{i}-\bar{x}\right){\left(x_{i}-\bar{x}\right)}^{T}$$

(5) $$S_{fb}=\sum_{j=1}^{c}\sum_{i=1}^{n}u_{ij}^{m}\left({\bar{x}}_{j}-\bar{x}\right){\left({\bar{x}}_{j}-\bar{x}\right)}^{T}$$

(6) $$S_{fw}=\sum_{j=1}^{c}\sum_{i=1}^{n}u_{ij}^{m}\left(x_{i}-{\bar{x}}_{j}\right){\left(x_{i}-{\bar{x}}_{j}\right)}^{T}$$

where m is the fuzzy weight index, and $u_{ij}$ represents the fuzzy membership (FM) value indicating the belongingness of the jth sample data to the ith class. For the calculation formula for $u_{ij}$, see Formula (4) in ref. [26].

#### 2.4.5. KNN

K-nearest neighbor (KNN) is one of the common classifiers and was used for the categorization of the chrysanthemum varieties in this experiment. As a supervised machine learning algorithm, its basic principle can be described as follows: Firstly, calculate the distances between a given test sample and each training sample. Then, find the K training samples with the closest distance, and, finally, predict the test sample class based on the class that occurs most frequently among the K samples.

PCA + LDA, PCA + IDLDA, and PCA + FIDLDA were used for extracting the discriminant information from the chrysanthemum tea samples’ spectra, and then the chrysanthemum tea varieties were classified by the KNN algorithm. The identification result of KNN is strongly related to the value of K. Therefore, the appropriate K was selected by computing the prediction accuracy under variant K values.

### 2.5. Software

In this study, the mathematics software we utilized was MATLAB (The Mathworks Inc., Natick, MA, USA) 2019a.

## 3. Results

### 3.1. NIR Spectral Analysis

The NIR spectra of the chrysanthemum tea samples in this experiment were within the wavelength range of 900–1700 nm. The original NIR spectra of the samples are shown in Figure 1, and the NIR spectra encompassed a large amount of information about molecular bonding and characteristic functional groups, such as C-H, O-H and N-H, which are likely to be associated with flavonoids, amino acids, and polysaccharides [43]. The absorption regions observed in the NIR spectra primarily originated from the band of groups containing hydrogen and its overtones. In Figure 1, the absorption bands are mainly concentrated in three regions, 1350 nm to 1370 nm, 1400 nm to 1470 nm, and 1630 nm to 1660 nm, respectively. From 920 nm to 940 nm, weak absorption bands can also be observed. The absorbance of the chrysanthemum tea dramatically changes after 1300 nm and reaches a peak at 1354 nm. This phenomenon may be related to the stretching vibration of the C-H and O-H groups in the amino acids and polysaccharides [44]. The absorption bands from 1400 nm to 1470 nm are ascribed to the first overtone of the O-H stretching vibrations alongside the N-H band [37]. The peak at 1652 nm is due to the C-H stretching first overtone of -CH_2_ and the binary combination bands involving C-H stretching modes [45,46].

### 3.2. Spectral Preprocessing

Figure 2 shows the NIR spectra of the chrysanthemum tea samples by different preprocessing methods. In this study, four single preprocessing methods were utilized: SNV, SG filtering, MSC, and MC, as well as two combined pretreatment methods, namely, SG + SNV and SG + MSC. The NIR spectra preprocessed by MC have no evident troughs and peaks in Figure 2b compared with the other spectra. We conducted experiments using six different pretreatment methods on the NIR spectra. Among them, it was observed that SG filtering had the best preprocessing effect, while the accuracy of the two mixed pretreatment methods combined with the proposed system was only about 80%, so we chose SG filtering as the preprocessing method in this study.

### 3.3. Dimensionality Reduction by PCA

**Figure 3:**
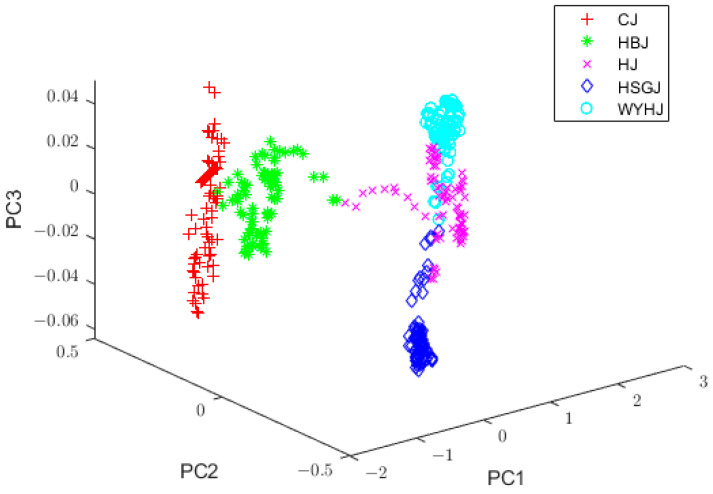
Distribution plot of vectors with PC1, PC2, and PC3 after SG filtering + PCA. PC1, PC2, and PC3, the first three principal components; SG, Savitsky–Golay; PCA, principal component analysis.

After preprocessing, the spectral data contained some redundant information and had high dimensionality. Such data were not conducive to the classification of the chrysanthemum tea varieties. Hence, it was essential to use PCA to extract the principal components (PCs) and mitigate redundant information. In this study, the total contribution of the first six PCs exceeded 99.98%, which proved that they retained the vast majority of the features in the NIR spectral data and eliminated a substantial quantity of redundant information. To be specific, the first six eigenvalues were listed as follows: $\lambda_{1}=552.9266$, $\lambda_{2}=25.0565$, $\lambda_{3}=0.3454$, $\lambda_{4}=0.1449$, $\lambda_{5}=0.0371$, and $\lambda_{6}=0.0182$. Hence, the 400-dimensional NIR spectra were projected into a six-dimensional feature space. Since the total contribution of the first three PCs reached 99.9%, a three-dimensional feature space was established to observe the distribution of the spectral data of the different kinds of chrysanthemum tea samples. Due to the four preprocessing methods used in this experiment, the spectral data obtained after the PCA processing were different. The distribution of the spectra processed by SG filtering and PCA in the three-dimensional feature space is shown in Figure 3, and it can be seen that the clustering of the data of the different kinds of samples is distinct, thus proving that PCA can effectively improve NIR spectral data. In addition, it is easy to see that the data after dimensionality reduction using PCA alone were still not good enough to identify the chrysanthemum tea samples, so more feature information needed to be extracted.

The subsequent sections cover the discussion of classification models, namely, PCA + LDA, PCA + IDILDA, and PCA + FIDILDA, applied to different chrysanthemum tea varieties.

### 3.4. Extraction of Features by LDA

**Figure 4:**
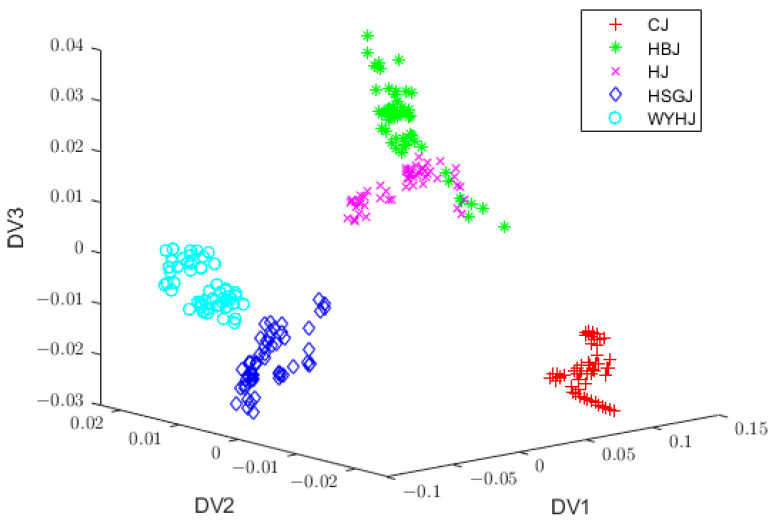
Three-dimensional data distribution by S-G filter + PCA + LDA. S-G, Savitsky–Golay; PCA, principal component analysis; LDA, linear discriminant analysis.

Following the PCA dimensionality reduction process, the 400 chrysanthemum tea samples were partitioned into a training set, which comprised 55 training samples for each variety (totaling 275 samples), and a test set containing 25 test samples for each variety (totaling 125 samples). The LDA algorithm was utilized for feature information extraction from the training set, and, subsequently, the test samples were projected onto the eigenvectors generated by the LDA. The rank of the inter-class scatter matrix was maximized by the number of classes minus one, so the number of eigenvectors and eigenvalues was four. Those four eigenvalues were listed as follows: $\lambda_{1}=64.5485$, $\lambda_{2}=16.2678$, $\lambda_{3}=12.1063$, and $\lambda_{4}=4.9531$. The six-dimensional feature data were projected onto the first three eigenvectors (DV1, DV2, and DV3) of the LDA, and the three-dimensional data distribution is shown in Figure 4. It is clear that PCA + LDA could distinguish the sample varieties to some extent, but there were two varieties of chrysanthemum tea sample data (HJ and HBJ) that overlapped with each other, and its classification accuracy was 87.2%. Therefore, a more effective feature extraction algorithm was imperative to improve the accuracy of the sample classification.

### 3.5. Discriminant Feature Extraction by IDLDA

**Figure 5:**
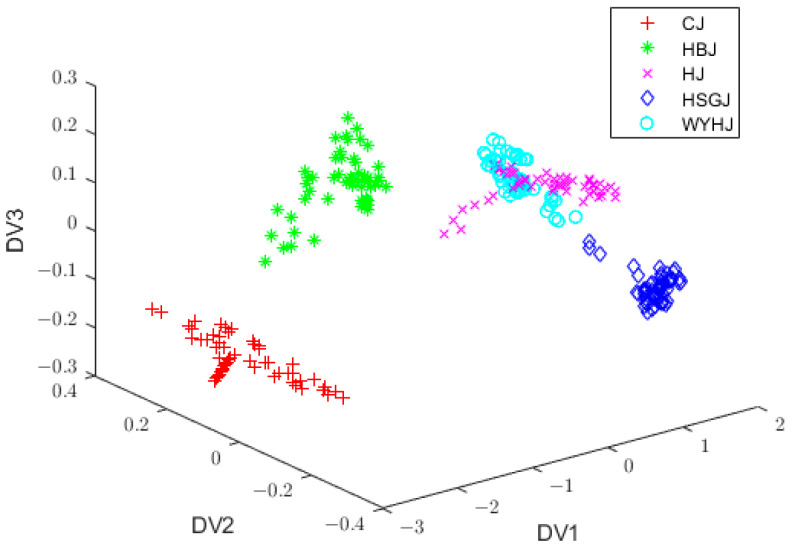
Three-dimensional data distribution by SG filtering + PCA + IDLDA. SG, Savitsky–Golay; PCA, principal component analysis; IDLDA, improved direct linear discriminant analysis.

After IDLDA extracted feature discriminative vectors from the six-dimensional data, it produced four discriminative vectors after processing the 275 training sets, and the PCA-processed data of the training samples were projected onto the first three discriminative vectors (DV1, DV2, and DV3). Figure 5 shows the scores plot of three discriminant eigenvectors of the IDLDA, and it can be seen that each sample datum had a more pronounced boundary profile. However, there was still some overlap between the two samples (HJ and WYHJ). Nevertheless, compared with the PCA + LDA algorithm, its classification accuracy was improved to 94.4%.

### 3.6. Feature Extraction by FIDLDA

FIDLDA performed feature extraction to transform the data into a feature space where the data were correctly classified. The results show that FIDLDA could address the limitations of IDLDA and improve the classification accuracy. All of the parameters related to FIDLDA were listed: the fuzzy weighting factor m=1.6 and the number of sample varieties c=5. The initial cluster center was represented by the mean of each variety of the chrysanthemum tea samples, and it is shown in Equation (7).

(7) $$V^{\left(0\right)}=\left(\begin{array}{c} {v_{1}}^{\left(0\right)}\\ {v_{2}}^{\left(0\right)}\\ {v_{3}}^{\left(0\right)}\\ {v_{4}}^{\left(0\right)}\\ {v_{5}}^{\left(0\right)} \end{array}\right)={\left(\begin{array}{rrrrrr} -1.1010 & 0.2820 & 0.0042 & -0.0290 & 0.0032 & -2.6439\\ -1.1897 & 0.0823 & 0.0063 & 0.0344 & 0.0035 & -4.7939\\ 0.0358 & -0.3044 & 0.0247 & -0.0069 & -0.0121 & 0.0040\\ 0.3013 & -0.1799 & -0.0493 & -0.0016 & 0.0029 & -0.0021\\ 1.6900 & 0.1031 & 0.0159 & -4.3758 & 0.0070 & -0.0011 \end{array}\right)}_{5\times 6}$$

**Figure 6:**
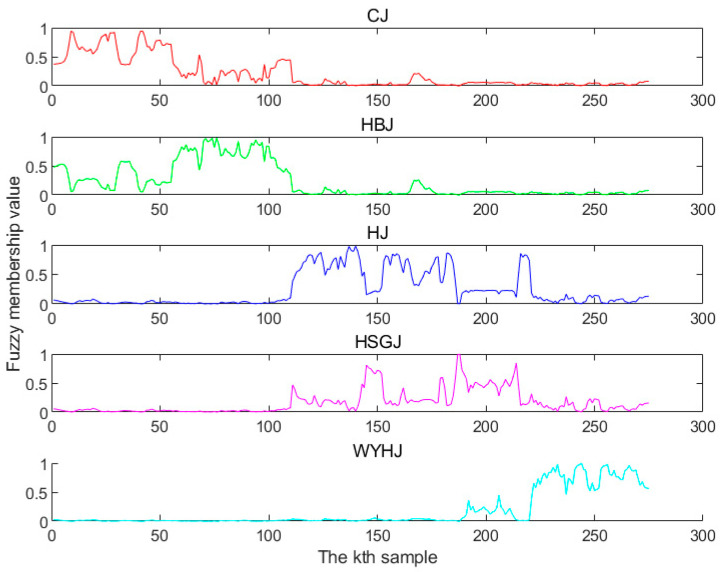
Initial fuzzy membership degrees.

Figure 6 displays the initial FM values, where the horizontal coordinate represents the chrysanthemum tea training sample and the vertical coordinate represents the FM values. Each little figure represents one chrysanthemum tea variety, namely, CJ, HBJ, HJ, HSGJ, and WYHJ, so there is a total of five little figures. If the FM degree of the *k*th sample was found to be the highest one within the jth category, it could be determined that the *k*th sample was attached to the corresponding jth category. The FM values of the HJ and HSGJ samples partially overlapped, which was due to calculating the FM values with the means of the sample data. Figure 3 shows that the score plots of HJ and HSGJ overlapped after PCA pretreatment, indicating that the means of two sample varieties were near, which negatively affected the calculation of the FM degrees.

**Figure 7:**
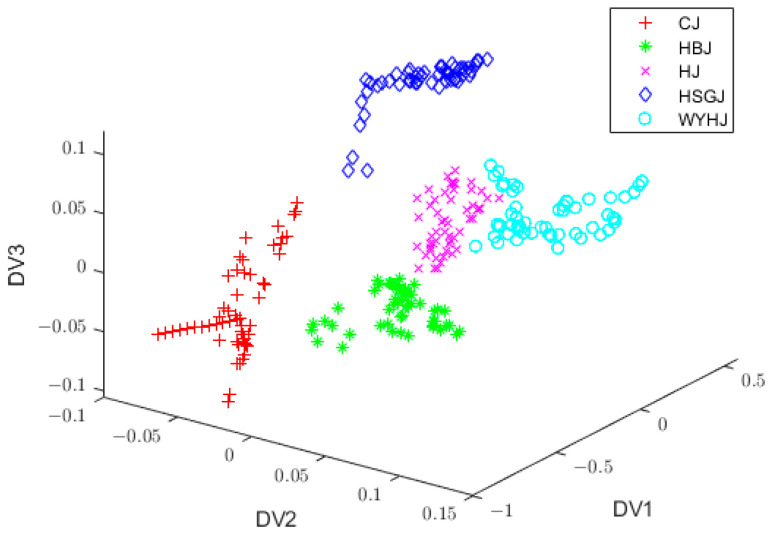
Three-dimensional data distribution by SG filtering + PCA + FIDLDA. S-G, Savitsky–Golay; PCA, principal component analysis; FIDLDA, fuzzy improved direct linear discriminant analysis.

Figure 7 displays the three-dimensional data distribution by SG filtering + PCA + FIDLDA. It can be seen that the samples of HJ and HSGJ were well separated, which indicated that FIDLDA significantly improved the recognition ability compared with LDA and IDLDA.

### 3.7. Classification Results of KNN

**Figure 8:**
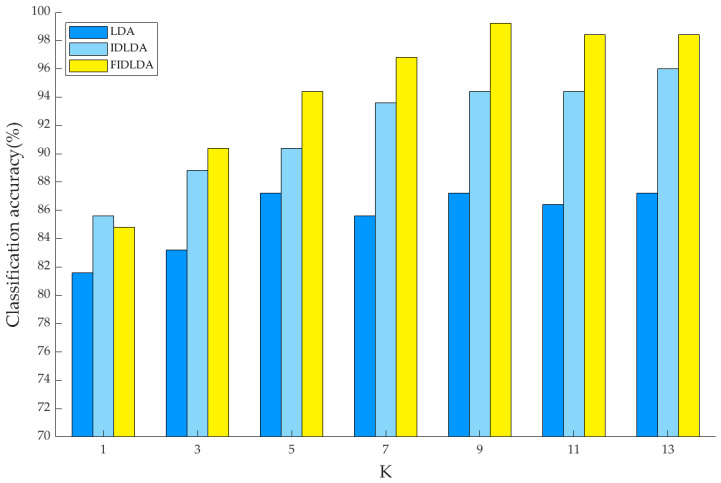
Classification accuracies of LDA, IDLDA, and FIDLDA with different K-values. LDA, linear discriminant analysis; IDLDA, improved direct linear discriminant analysis; FIDLDA, fuzzy improved direct linear discriminant analysis.

The KNN algorithm was employed as a classifier for the identification of the chrysanthemum tea varieties in the data after using the feature extraction algorithms. Since the K-value can affect the classification accuracy of KNN, in order to obtain the K-value for optimal identification accuracy, we employed KNN using different K-values (1, 3, 5, 7, 9, 11, and 13) with three feature extraction methods (LDA, IDLDA, and FIDLDA) for the calculation of the prediction accuracy. The training sample set consisted of 275 samples, and the test sample set comprised 125 samples. The classification accuracies with different K-values are shown in Figure 8. In comparison with LDA and IDLDA, the FIDLDA algorithm had the highest classification accuracy of 99.2% when the value of K was nine. Thus, it was proved that the FIDLDA algorithm combined with the KNN classifier had a great classification ability.

## 4. Discussion

Firstly, the NIR spectra of chrysanthemum tea samples were obtained by a portable spectrometer, and then SG filtering was used for noise reduction, PCA for data dimensionality reduction, and LDA, IDLDA, and FIDLDA for feature information extraction. Finally, KNN was utilized as a classifier to categorize the sample varieties. In Figure 8, it is obvious that using different feature extraction algorithms obtained different classification accuracies. When the traditional LDA algorithm was employed to extract features, the classification accuracy was below 90%. In comparison, when the FIDLDA was applied as a feature extraction algorithm, the highest identification accuracy achieved a value of 99.2%.

**Figure 9:**
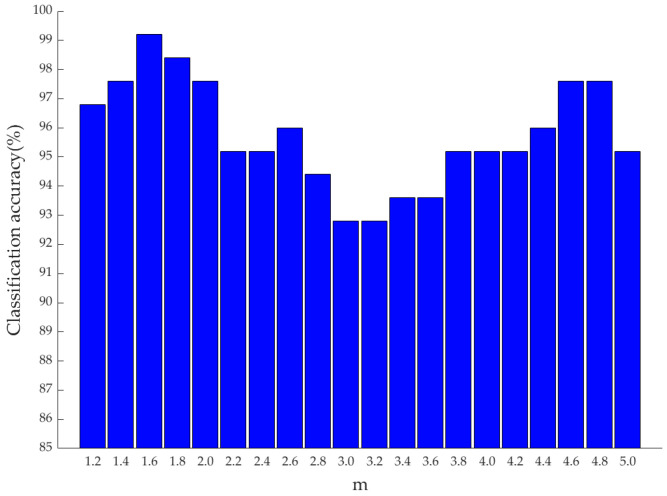
Classification accuracies of FIDLDA with different values of fuzzy weight index m. FIDLDA, fuzzy improved direct linear discriminant analysis.

The fuzzy weight index m has a strong correlation with the feature extraction effect of FIDLDA. We conducted the experiments using different values of m and recorded the classification accuracies accordingly. In particular, the value of m could not be lower than 1, so *m* ranged between 1.2 and 5.0. Figure 9 shows the classification accuracy of FIDLDA with different m-values, and it reached the highest classification accuracy when the value of m was 1.6.

The data quantities in the training set and test set also affect the classification accuracy of the classification model. Other things being equal, we observed the classification accuracies obtained from three different combinations of training and test samples. Table 2 shows the categorization accuracies using LDA, IDLDA, and FIDLDA with different data quantities for the training and test sets for the chrysanthemum tea varieties. Table 2 shows that FIDLDA produced higher classification accuracies than LDA and IDLDA. When the data quantities for the training set and test set were 275 and 125, respectively, the FIDLDA algorithm reached the highest accuracy of 99.20%.

To show the superiority of the FIDLDA model for chrysanthemum tea varieties, the FIPLDA-KNN model and the FIPLDA-SVM model [44], which have been applied for chrysanthemum tea identification, were used for comparison. When the S-G filtering algorithm was also used for preprocessing, and PCA was used for dimensionality reduction, the FIPLDA-KNN model achieved the maximum classification accuracy of 98.33% when the fuzzy weight coefficient was 2.7 and K was 7, while the FIPLDA-SVM model had the maximum classification accuracy of 90.83%. The specific results can be found in ref. [44]. In contrast, the FIDLDA model reached a classification accuracy of 99.2% in the identification of chrysanthemum tea. Therefore, the proposed nondestructive discrimination system for chrysanthemum tea varieties in this study had a better performance than the models used in the previous research.

## 5. Conclusions

In order to be able to quickly, non-destructively, and effectively discriminate chrysanthemum tea varieties, a classification system combining NIR spectroscopy with the FIDLDA algorithm was presented in this study. The proposed FIDLDA algorithm is a unique fusion of the fuzzy set and the IDLDA algorithm, and it provides a novel approach for extracting features from chrysanthemum tea spectral data after PCA reduces the data dimensionality. At first, the NIR spectra of the chrysanthemum tea samples were acquired by a portable spectrometer. Secondly, SG filtering, PCA, LDA, IDLDA, and FIDLDA were utilized for data denoising, dimensional reduction, and feature extraction from the data, respectively. Finally, the KNN algorithm was used to classify the chrysanthemum tea varieties.

The results show that the FIDLDA algorithm had the highest accuracy in the classification of chrysanthemum tea varieties compared with the LDA and IDLDA algorithms. This study illustrates that the combination of NIR spectroscopy and the FIDLDA algorithm has great potential for the nondestructive discrimination of chrysanthemum tea varieties.

## Figures and Tables

**Table 1:** The contents of several functional components of five varieties of chrysanthemum tea (%).

	CJ	HBJ	HJ	HSGJ	WYHJ
Flavone	16.08	14.22	10.25	13.39	4.13
Soluble sugar	23.18	16.35	19.04	23.05	15.01
Chlorogenic acid	3.75	3.47	2.16	1.09	-

**Table 2:** Classification accuracies of LDA, IDLDA, and FIDLDA with different n_training and n_test combinations (%).

n_training	n_test	LDA	IDLDA	FIDLDA
250	150	87.33	94.00	95.33
275	125	87.20	94.40	99.20
300	100	90.00	91.00	95.00

Abbreviations: LDA, linear discriminant analysis; IDLDA, improved direct linear discriminant analysis; FIDLDA, fuzzy improved direct linear discriminant analysis.

## Data Availability

The original contributions presented in the study are included in the article and supplementary materials, further inquiries can be directed to the corresponding author.
